# 2-Amino-5-methyl­pyridinium 2-carb­oxy­benzoate

**DOI:** 10.1107/S160053681003000X

**Published:** 2010-08-04

**Authors:** Madhukar Hemamalini, Hoong-Kun Fun

**Affiliations:** a X-ray Crystallography Unit, School of Physics, Universiti Sains Malaysia, 11800 USM, Penang, Malaysia

## Abstract

In the title salt, C_6_H_9_N_2_^+^·C_8_H_5_O_4_^−^, the hydrogen phthalate anion is essentially planar, with a maximum deviation of 0.011 (2) Å. In the crystal structure, the protonated N atom of the pyridine ring and the 2-amino group of the cation are hydrogen bonded to the carboxyl­ate O atoms of the anion *via* a pair of N—H⋯O hydrogen bonds, forming an *R*_2_^2^(8) ring motif. In the hydrogen phthalate anion, there is a very strong, almost symmetric, intra­molecular O—H⋯O hydrogen bond, generating an *S*(7) motif [O⋯O = 2.382 (3) Å]. Furthermore, these two molecular motif rings are connected by a bifurcated N—H⋯(O,O) hydrogen-bonded motif *R*_1_^2^(4), forming a supra­molecular ribbon along the *b* axis. The crystal structure is further stabilized by π–π inter­actions between the cations and anions [centroid–centroid distance = 3.6999 (10) Å].

## Related literature

For the crystal structure of phthalic acid, see: Nowacki & Jaggi (1957 ▶); Küppers (1981 ▶); Ermer (1981 ▶). For the crystal structures of hydrogen phthalates, see: Jessen (1990 ▶); Jin *et al.* (2003 ▶); Küppers (1978 ▶). For a description of the Cambridge Structural Database, see: Allen (2002 ▶). For hydrogen-bond motifs, see: Bernstein *et al.* (1995 ▶). For reference bond-length data, see: Allen *et al.* (1987 ▶).
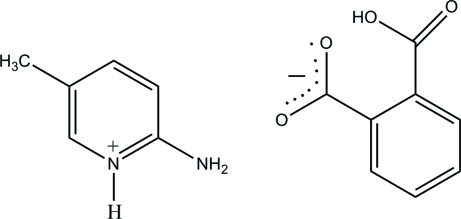


## Experimental

### 

#### Crystal data

C_6_H_9_N_2_^+^·C_8_H_5_O_4_^−^
*M* *_r_* = 274.27
Monoclinic, 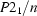

*a* = 11.3853 (2) Å
*b* = 8.8203 (2) Å
*c* = 13.4617 (3) Å
β = 101.540 (2)°
*V* = 1324.52 (5) Å^3^
*Z* = 4
Mo *K*α radiation
μ = 0.10 mm^−1^
*T* = 296 K
0.47 × 0.33 × 0.20 mm

#### Data collection

Bruker SMART APEXII CCD area-detector diffractometer
Absorption correction: multi-scan (*SADABS*; Bruker, 2009 ▶) *T*_min_ = 0.953, *T*_max_ = 0.980
12332 measured reflections
3049 independent reflections
2054 reflections with *I* > 2σ(*I*)
*R*_int_ = 0.028

#### Refinement

*R*[*F* ^2^ > 2σ(*F* ^2^)] = 0.049
*wR*(*F* ^2^) = 0.136
*S* = 1.03
3049 reflections
197 parameters
H atoms treated by a mixture of independent and constrained refinement
Δρ_max_ = 0.19 e Å^−3^
Δρ_min_ = −0.14 e Å^−3^

### 

Data collection: *APEX2* (Bruker, 2009 ▶); cell refinement: *SAINT* (Bruker, 2009 ▶); data reduction: *SAINT*; program(s) used to solve structure: *SHELXTL* (Sheldrick, 2008 ▶); program(s) used to refine structure: *SHELXTL*; molecular graphics: *SHELXTL*; software used to prepare material for publication: *SHELXTL* and *PLATON* (Spek, 2009 ▶).

## Supplementary Material

Crystal structure: contains datablocks global, I. DOI: 10.1107/S160053681003000X/wn2402sup1.cif

Structure factors: contains datablocks I. DOI: 10.1107/S160053681003000X/wn2402Isup2.hkl

Additional supplementary materials:  crystallographic information; 3D view; checkCIF report

## Figures and Tables

**Table 1:** Hydrogen-bond geometry (Å, °)

*D*—H⋯*A*	*D*—H	H⋯*A*	*D*⋯*A*	*D*—H⋯*A*
N1—H1*N*1⋯O2^i^	0.928 (19)	1.786 (19)	2.713 (2)	175.7 (17)
N2—H2*N*2⋯O1^i^	0.95 (2)	1.97 (2)	2.907 (3)	173 (2)
N2—H1*N*2⋯O3^ii^	0.90 (3)	2.39 (3)	3.161 (2)	143 (2)
N2—H1*N*2⋯O4^ii^	0.90 (3)	2.28 (3)	3.151 (3)	162 (2)
O1—H1*O*1⋯O3	1.16 (2)	1.22 (2)	2.382 (3)	175 (2)

Symmetry codes: (i) 
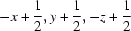
; (ii) 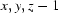.

**Fig. 1.**
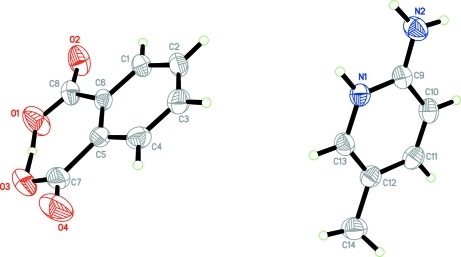
The asymmetric unit of the title compound. Displacement ellipsoids are drawn at the 50% probability level. Hydrogen atoms are shown as spheres of arbitrary radius. A solid line indicates the intramolecular hydrogen bond.

**Fig. 2.**
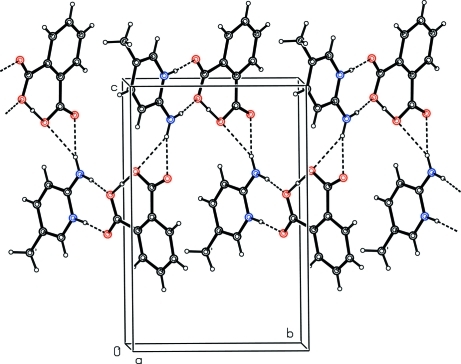
A view, down the a-axis, of supramolecular ribbons made up of cations and anions. The dashed lines indicate hydrogen bonds.

## supplementary crystallographic information

### Comment

The crystal structure of phthalic acid (Nowacki & Jaggi, 1957; Küppers, 1981; Ermer, 1981) has been reported several times. Analysis of the structures archived in the Cambridge Structural Database (Version 5.28; Allen, 2002) shows that the hydrogen phthalate ions of phthalate salts occur in two different forms: (i) non-planar, where both the carboxyl (COOH) and the carboxylate (COO^-^) groups are twisted out of the plane of the benzene ring (Jessen, 1990; Jin *et al.*, 2003), and (ii) planar, in which both the COOH and the COO^-^ groups are coplanar with the benzene plane (Küppers, 1978).  Since our aim is to study some interesting hydrogen-bonding interactions, the crystal structure of the title compound is presented here.

The asymmetric unit of the title compound (Fig 1), contains a protonated 2-amino-5-methylpyridinium cation and a hydrogen phthalate anion. In the 2-amino-5-methylpyridinium cation, a wide angle (122.45 (16)°) is subtended at the protonated N1 atom. The hydrogen phthalate anion is almost planar, with a maximum deviation of 0.011 (2) Å for atom C5. The bond lengths are normal (Allen *et al.*, 1987).

In the crystal structure (Fig. 2), the protonated N1 atom and the 2-amino group (N2) are hydrogen-bonded to the carboxylate oxygen atoms (O1 and O2) via a pair of N—H···O (Table 1) hydrogen bonds forming an *R*_2_^2^(8) ring motif (Bernstein *et al.*, 1995). In the hydrogen phthalate anion, there is a very strong intramolecular O—H···O hydrogen bond, generating an *S*(7) motif [O1···O3 =  2.382 (3) Å]. Furthermore, these two molecular motif rings are connected by a bifurcated hydrogen bonded motif *R*^2^_1_(4), involving H1N2 of the 2-amino group and carboxyl atoms O3 and O4 to form a supramolecular ribbon along the *b*-axis. The crystal structure is further stabilized by π–π interactions between the cations and anions [centroid-centroid distance = 3.6999 (10) Å ; symmetry code: -1/2+x, 1/2-y, 1/2+z].

### Experimental

A hot methanol solution (20 ml) of 2-amino-5-methylpyridine (27 mg, Aldrich) and phthalic acid (41 mg, Merck) were mixed and warmed over a heating magnetic stirrer hotplate for a few minutes. The resulting solution was allowed to cool slowly at room temperature and crystals of the title compound appeared after a few days.

### Refinement

Atoms H1N1, H2N2, H1N2 and H1O1 were located from a difference Fourier map and were refined freely [N—H= 0.90 (3) – 0.95 (2) Å and O—H = 1.16 (3) – 1.22 (3) Å]. The remaining hydrogen atoms were positioned geometrically [C—H = 0.93 or 0.96 Å] and were refined using a riding model, with *U*_iso_(H) = 1.2 or 1.5*U*_eq_(C). A rotating group model was used for the methyl group.

### Crystal data

**Table:**

C_6_H_9_N_2_^+^·C_8_H_5_O_4_^−^	*F*(000) = 576
*M**_r_* = 274.27	*D*_x_ = 1.375 Mg m^−^^3^
Monoclinic, *P*2_1_/*n*	Mo *K*α radiation, λ = 0.71073 Å
Hall symbol: -P 2yn	Cell parameters from 3124 reflections
*a* = 11.3853 (2) Å	θ = 2.6–28.6°
*b* = 8.8203 (2) Å	µ = 0.10 mm^−^^1^
*c* = 13.4617 (3) Å	*T* = 296 K
β = 101.540 (2)°	Block, colourless
*V* = 1324.52 (5) Å^3^	0.47 × 0.33 × 0.20 mm
*Z* = 4	

### Data collection

**Table:**

Bruker SMART APEXII CCD area-detector diffractometer	3049 independent reflections
Radiation source: fine-focus sealed tube	2054 reflections with *I* > 2σ(*I*)
graphite	*R*_int_ = 0.028
φ and ω scans	θ_max_ = 27.5°, θ_min_ = 2.6°
Absorption correction: multi-scan (*SADABS*; Bruker, 2009)	*h* = −14→14
*T*_min_ = 0.953, *T*_max_ = 0.980	*k* = −11→11
12332 measured reflections	*l* = −17→12

### Refinement

**Table:**

Refinement on *F*^2^	Primary atom site location: structure-invariant direct methods
Least-squares matrix: full	Secondary atom site location: difference Fourier map
*R*[*F*^2^ > 2σ(*F*^2^)] = 0.049	Hydrogen site location: inferred from neighbouring sites
*wR*(*F*^2^) = 0.136	H atoms treated by a mixture of independent and constrained refinement
*S* = 1.03	*w* = 1/[σ^2^(*F*_o_^2^) + (0.0585*P*)^2^ + 0.230*P*] where *P* = (*F*_o_^2^ + 2*F*_c_^2^)/3
3049 reflections	(Δ/σ)_max_ < 0.001
197 parameters	Δρ_max_ = 0.19 e Å^−^^3^
0 restraints	Δρ_min_ = −0.14 e Å^−^^3^

### Special details

**Table:**

Geometry. All esds (except the esd in the dihedral angle between two l.s. planes) are estimated using the full covariance matrix. The cell esds are taken into account individually in the estimation of esds in distances, angles and torsion angles; correlations between esds in cell parameters are only used when they are defined by crystal symmetry. An approximate (isotropic) treatment of cell esds is used for estimating esds involving l.s. planes.
Refinement. Refinement of F^2^ against ALL reflections. The weighted R-factor wR and goodness of fit S are based on F^2^, conventional R-factors R are based on F, with F set to zero for negative F^2^. The threshold expression of F^2^ > 2sigma(F^2^) is used only for calculating R-factors(gt) etc. and is not relevant to the choice of reflections for refinement. R-factors based on F^2^ are statistically about twice as large as those based on F, and R- factors based on ALL data will be even larger.

### Fractional atomic coordinates and isotropic or equivalent isotropic displacement parameters (Å^2^)

**Table:**

	*x*	*y*	*z*	*U*_iso_*/*U*_eq_	
O1	0.22365 (18)	−0.0844 (2)	0.56803 (13)	0.1122 (6)	
O2	0.11271 (15)	−0.09799 (18)	0.41687 (11)	0.0907 (5)	
O3	0.39091 (17)	0.0516 (2)	0.66089 (11)	0.1042 (6)	
O4	0.51382 (17)	0.2270 (2)	0.63733 (13)	0.1129 (6)	
C1	0.24378 (17)	0.09471 (19)	0.33146 (12)	0.0560 (4)	
H1A	0.1762	0.0456	0.2956	0.067*	
C2	0.30478 (17)	0.1928 (2)	0.28048 (13)	0.0609 (5)	
H2A	0.2777	0.2107	0.2116	0.073*	
C3	0.40493 (17)	0.2635 (2)	0.33145 (15)	0.0689 (5)	
H3A	0.4481	0.3277	0.2973	0.083*	
C4	0.44221 (17)	0.2395 (2)	0.43406 (16)	0.0675 (5)	
H4A	0.5104	0.2894	0.4682	0.081*	
C5	0.38123 (16)	0.14284 (19)	0.48854 (12)	0.0547 (4)	
C6	0.27973 (15)	0.06614 (17)	0.43513 (12)	0.0508 (4)	
C7	0.4338 (2)	0.1418 (3)	0.60205 (16)	0.0719 (6)	
C8	0.1997 (2)	−0.0461 (2)	0.47582 (15)	0.0644 (5)	
N1	0.55034 (14)	0.21351 (16)	0.02651 (10)	0.0539 (4)	
N2	0.46340 (18)	0.2514 (2)	−0.14144 (14)	0.0712 (5)	
C9	0.54553 (16)	0.18327 (18)	−0.07217 (12)	0.0532 (4)	
C10	0.62974 (17)	0.0798 (2)	−0.09620 (14)	0.0611 (5)	
H10A	0.6290	0.0552	−0.1635	0.073*	
C11	0.71156 (17)	0.0164 (2)	−0.02130 (14)	0.0619 (5)	
H11A	0.7671	−0.0511	−0.0383	0.074*	
C12	0.71554 (16)	0.04912 (19)	0.08173 (13)	0.0573 (4)	
C13	0.63244 (16)	0.14845 (19)	0.10155 (13)	0.0581 (4)	
H13A	0.6316	0.1729	0.1686	0.070*	
C14	0.80669 (19)	−0.0225 (3)	0.16415 (16)	0.0798 (6)	
H14A	0.7953	0.0133	0.2289	0.120*	
H14B	0.8858	0.0039	0.1553	0.120*	
H14C	0.7975	−0.1307	0.1611	0.120*	
H1N1	0.4931 (17)	0.279 (2)	0.0425 (14)	0.067 (6)*	
H2N2	0.404 (2)	0.312 (3)	−0.1211 (17)	0.086 (7)*	
H1N2	0.466 (2)	0.227 (3)	−0.206 (2)	0.096 (8)*	
H1O1	0.308 (2)	−0.022 (3)	0.6119 (18)	0.100 (7)*	

### Atomic displacement parameters (Å^2^)

**Table:**

	*U*^11^	*U*^22^	*U*^33^	*U*^12^	*U*^13^	*U*^23^
O1	0.1250 (15)	0.1378 (15)	0.0725 (10)	−0.0338 (12)	0.0163 (10)	0.0395 (10)
O2	0.1126 (12)	0.0943 (11)	0.0705 (9)	−0.0427 (9)	0.0308 (9)	−0.0029 (8)
O3	0.1091 (13)	0.1526 (16)	0.0486 (8)	0.0056 (12)	0.0099 (9)	0.0157 (10)
O4	0.1112 (14)	0.1306 (15)	0.0794 (11)	−0.0074 (12)	−0.0228 (10)	−0.0167 (10)
C1	0.0660 (11)	0.0552 (9)	0.0472 (9)	−0.0033 (8)	0.0125 (8)	−0.0049 (7)
C2	0.0717 (12)	0.0679 (11)	0.0459 (9)	0.0020 (9)	0.0182 (8)	0.0024 (8)
C3	0.0639 (12)	0.0761 (12)	0.0709 (12)	−0.0040 (10)	0.0236 (10)	0.0115 (10)
C4	0.0502 (10)	0.0771 (12)	0.0731 (13)	−0.0024 (9)	0.0072 (9)	−0.0031 (10)
C5	0.0560 (10)	0.0579 (9)	0.0500 (9)	0.0147 (8)	0.0100 (8)	−0.0032 (7)
C6	0.0617 (10)	0.0460 (8)	0.0478 (9)	0.0080 (7)	0.0185 (8)	−0.0019 (7)
C7	0.0707 (13)	0.0821 (14)	0.0587 (11)	0.0237 (11)	0.0026 (10)	−0.0087 (10)
C8	0.0841 (14)	0.0567 (10)	0.0575 (11)	−0.0010 (9)	0.0265 (10)	0.0015 (8)
N1	0.0587 (9)	0.0533 (8)	0.0517 (8)	0.0012 (7)	0.0158 (7)	−0.0046 (6)
N2	0.0868 (13)	0.0727 (11)	0.0517 (10)	0.0110 (9)	0.0079 (9)	−0.0033 (8)
C9	0.0635 (11)	0.0470 (8)	0.0502 (9)	−0.0094 (8)	0.0140 (8)	−0.0026 (7)
C10	0.0753 (12)	0.0588 (10)	0.0532 (10)	−0.0047 (9)	0.0225 (9)	−0.0102 (8)
C11	0.0617 (11)	0.0564 (10)	0.0710 (11)	0.0005 (8)	0.0216 (9)	−0.0101 (9)
C12	0.0543 (10)	0.0569 (9)	0.0613 (10)	−0.0037 (8)	0.0133 (8)	−0.0035 (8)
C13	0.0642 (11)	0.0639 (10)	0.0468 (9)	−0.0019 (9)	0.0124 (8)	−0.0036 (8)
C14	0.0699 (13)	0.0880 (14)	0.0781 (13)	0.0120 (11)	0.0064 (11)	−0.0013 (11)

### Geometric parameters (Å, °)

**Table:**

O1—C8	1.263 (2)	N1—C9	1.345 (2)
O1—H1O1	1.16 (3)	N1—C13	1.359 (2)
O2—C8	1.227 (2)	N1—H1N1	0.93 (2)
O3—C7	1.286 (3)	N2—C9	1.325 (2)
O3—H1O1	1.22 (3)	N2—H2N2	0.95 (2)
O4—C7	1.203 (3)	N2—H1N2	0.90 (3)
C1—C2	1.376 (2)	C9—C10	1.407 (3)
C1—C6	1.396 (2)	C10—C11	1.350 (3)
C1—H1A	0.9300	C10—H10A	0.9300
C2—C3	1.359 (3)	C11—C12	1.409 (2)
C2—H2A	0.9300	C11—H11A	0.9300
C3—C4	1.378 (3)	C12—C13	1.355 (2)
C3—H3A	0.9300	C12—C14	1.499 (3)
C4—C5	1.397 (3)	C13—H13A	0.9300
C4—H4A	0.9300	C14—H14A	0.9600
C5—C6	1.406 (2)	C14—H14B	0.9600
C5—C7	1.525 (3)	C14—H14C	0.9600
C6—C8	1.520 (3)		
			
C8—O1—H1O1	111.6 (12)	C9—N1—H1N1	117.5 (11)
C7—O3—H1O1	110.1 (11)	C13—N1—H1N1	120.1 (11)
C2—C1—C6	122.40 (17)	C9—N2—H2N2	119.9 (13)
C2—C1—H1A	118.8	C9—N2—H1N2	114.5 (15)
C6—C1—H1A	118.8	H2N2—N2—H1N2	125 (2)
C3—C2—C1	119.53 (17)	N2—C9—N1	119.25 (17)
C3—C2—H2A	120.2	N2—C9—C10	123.33 (17)
C1—C2—H2A	120.2	N1—C9—C10	117.42 (16)
C2—C3—C4	119.66 (18)	C11—C10—C9	119.81 (16)
C2—C3—H3A	120.2	C11—C10—H10A	120.1
C4—C3—H3A	120.2	C9—C10—H10A	120.1
C3—C4—C5	122.31 (18)	C10—C11—C12	122.15 (17)
C3—C4—H4A	118.8	C10—C11—H11A	118.9
C5—C4—H4A	118.8	C12—C11—H11A	118.9
C4—C5—C6	117.98 (16)	C13—C12—C11	116.12 (16)
C4—C5—C7	113.14 (18)	C13—C12—C14	122.30 (17)
C6—C5—C7	128.82 (17)	C11—C12—C14	121.58 (17)
C1—C6—C5	118.08 (16)	C12—C13—N1	122.04 (16)
C1—C6—C8	113.62 (16)	C12—C13—H13A	119.0
C5—C6—C8	128.29 (16)	N1—C13—H13A	119.0
O4—C7—O3	119.6 (2)	C12—C14—H14A	109.5
O4—C7—C5	120.4 (2)	C12—C14—H14B	109.5
O3—C7—C5	120.0 (2)	H14A—C14—H14B	109.5
O2—C8—O1	121.58 (19)	C12—C14—H14C	109.5
O2—C8—C6	118.33 (16)	H14A—C14—H14C	109.5
O1—C8—C6	120.09 (19)	H14B—C14—H14C	109.5
C9—N1—C13	122.45 (16)		
			
C6—C1—C2—C3	1.1 (3)	C1—C6—C8—O2	−1.1 (2)
C1—C2—C3—C4	−1.9 (3)	C5—C6—C8—O2	178.03 (17)
C2—C3—C4—C5	0.7 (3)	C1—C6—C8—O1	178.51 (19)
C3—C4—C5—C6	1.2 (3)	C5—C6—C8—O1	−2.3 (3)
C3—C4—C5—C7	−176.18 (17)	C13—N1—C9—N2	−179.69 (16)
C2—C1—C6—C5	0.8 (3)	C13—N1—C9—C10	0.1 (2)
C2—C1—C6—C8	−179.89 (16)	N2—C9—C10—C11	179.23 (17)
C4—C5—C6—C1	−1.9 (2)	N1—C9—C10—C11	−0.5 (3)
C7—C5—C6—C1	175.00 (16)	C9—C10—C11—C12	0.6 (3)
C4—C5—C6—C8	178.91 (16)	C10—C11—C12—C13	−0.1 (3)
C7—C5—C6—C8	−4.1 (3)	C10—C11—C12—C14	179.80 (18)
C4—C5—C7—O4	5.3 (3)	C11—C12—C13—N1	−0.3 (3)
C6—C5—C7—O4	−171.79 (19)	C14—C12—C13—N1	179.74 (17)
C4—C5—C7—O3	−176.07 (19)	C9—N1—C13—C12	0.4 (3)
C6—C5—C7—O3	6.9 (3)		

### Hydrogen-bond geometry (Å, °)

**Table:**

*D*—H···*A*	*D*—H	H···*A*	*D*···*A*	*D*—H···*A*
N1—H1N1···O2^i^	0.928 (19)	1.786 (19)	2.713 (2)	175.7 (17)
N2—H2N2···O1^i^	0.95 (2)	1.97 (2)	2.907 (3)	173 (2)
N2—H1N2···O3^ii^	0.90 (3)	2.39 (3)	3.161 (2)	143 (2)
N2—H1N2···O4^ii^	0.90 (3)	2.28 (3)	3.151 (3)	162 (2)
O1—H1O1···O3	1.16 (2)	1.22 (2)	2.382 (3)	175 (2)

Symmetry codes: (i) −*x*+1/2, *y*+1/2, −*z*+1/2; (ii) *x*, *y*, *z*−1.
